# Water Researchers Do Not Have a Strategic Plan for Gathering Evidence to Inform Water Intake Recommendations to Prevent Chronic Disease

**DOI:** 10.3390/nu12113359

**Published:** 2020-10-31

**Authors:** Jodi D. Stookey, Stavros A. Kavouras

**Affiliations:** Hydration Science Laboratory, College of Health Solutions, Arizona State University, Phoenix, AZ 85004, USA; stavros.kavouras@asu.edu

**Keywords:** water intake, hydration, serum osmolality, hypernatremia, urine osmolality, dietary recommendations, chronic disease, strategic planning

## Abstract

Confusion has persisted for decades in the United States (U.S.) over how much plain water to drink, despite national water intake recommendations which are based on high quality scientific evidence. This editorial summarizes the definition, alignment and coordination of evidence that informs the current U.S. adequate intake (AI) recommendations for water. It highlights gaps in the evidence that perpetuate confusion and opportunity to address the gaps through strategic planning.

## 1. Confusion about How Much Water to Drink Has Persisted for Decades

In 1945, the U.S. Food and Nutrition Board of the National Research Council published a water intake recommendation of 1 mL/kcal energy expended [1]. For a person who is in energy balance with a usual total energy intake of 2000 kcal/d, this recommendation translates to 2 L/d or 64 oz, which is equivalent to eight 8 oz glasses. Although the recommendation refers to intake of total water, i.e., water from all food and beverage sources combined, the equivalency with eight 8 oz glasses appears to have been confused with advice to *drink* eight 8 oz glasses of *plain water* each day [2,3,4].

Two decades ago, researchers noted the difference between 2 L/d total water and 2 L/d plain water and cautioned that available evidence did not support advice to drink “8 × 8” glasses of water [4,5]. Lindeman et al. [5] argued that intake of eight 8 oz glasses of plain water exceeds the volume needed by older adults because, in their experience, healthy older adults who consume less than 6 oz of fluid per day do not have hypernatremic dehydration. Valtin [4] voiced similar concern about lack of evidence for 8 × 8 glasses of plain water and potential for harm related to incontinence and hyponatremia. The paper by Valtin [4] was widely publicized. CBS News reported, for example, that “trying to do the ‘right’ thing by drinking eight full glasses of water a day may do little more than make a person run to the bathroom.” [6] A 2004 WHO rolling revision report suggests that “unsubstantiated claims about the essentiality of plain water” for meeting fluid requirements shaped public perception in the U.S. [7].

Public confusion about how much plain water to drink continues to the present day. In 2015, for example, the New York Times ran an article entitled “No, You Do Not Have to Drink 8 Glasses of Water a Day” [8]. In May of 2020, a BBC.com article entitled “How much water should you drink a day?” noted that “advice comes from decade-old guidance” and “may have no scientific basis” [9].

This editorial calls for strategic planning to address lingering questions about how much plain water to drink to avoid acute and chronic health conditions. It focuses on how much plain water to drink because the lingering confusion implies that the public expects a recommendation for water, such as what is available for other nutrients like vitamin C. For vitamin C, individuals can choose to attend to their requirement by consuming foods or beverages and/or consuming the nutrient in pill form [10]. This editorial focuses on chronic health conditions because they are the leading causes of death in the U.S. and their prevention is a U.S. public health priority [11].

## 2. U.S. Water Intake Recommendations Are Evidence-Based

Despite persistent concern that the “8 × 8” recommendation lacks scientific backing, the official U.S. water intake recommendations are indisputably evidence-based. The current adequate intake (AI) recommendations for water for the U.S., released in 2005, by the Institute of Medicine (now the National Academies of Medicine (NAM)) [12], reflect elegant definition, alignment, and coordination of experimental and observational evidence (see Table 1).

### 2.1. Definition

To develop the AI recommendations for water, the NAM convened a committee of experts that set a high standard of evidence for the recommendations. They defined health impact in terms of experimental evidence and restricted the scope of the 2005 AI for water to acute health effects only because controlled experiments addressing chronic disease were lacking [12].

“evidence is insufficient to establish water intake recommendations as a means to reduce the risk of chronic diseases. Instead an Adequate Intake (AI) for total water is set to prevent deleterious, primarily acute, effects of dehydration, which include metabolic and functional abnormalities.”

[12] (p. 93)

The NAM report [12] defines hydration in terms of total body water (TBW) and documents working assumption that chronic TBW deficit does not occur under conditions of normal daily life in the U.S. This assumption, in turn, enables further assumption that the estimated total water intake (TWI) of the U.S. population can serve as proxy for the unmeasured, underlying total water requirement. The underlying total water requirement is difficult to measure because myriad factors which determine water requirement, including sex, age, body size, health status, solute intake, physical activity, and environment, vary between individuals.

“Daily water intake must be balanced with losses in order to maintain total body water. Body water deficits challenge the ability to maintain homeostasis during perturbations (e.g., sickness, physical exercise, and environmental exposure) and can affect function and health.”

[12] (p. 94)

“Over the course of a few hours, body water deficits can occur due to reduced intake or increased water losses from physical activity and environmental (e.g., heat) exposure. However, on a day to day basis, fluid intake, driven by the combination of thirst and the consumption of beverages at meals, allows maintenance of hydration status and total body water at normal levels.”

[12] (p. 74)

“Water balance is regulated within ±0.2 percent of body weight over a 24-h period for healthy adults at rest (Adolph, 1943)… Newburg and colleagues (1930) demonstrated the accuracy of water balance studies to be within 0.5 percent of the water volume. Therefore, ad-libitum water balance studies can be used to estimate daily water requirements, provided the subjects have adequate time for rehydration and physiologic compensation.”

[12] (p. 86)

The NAM report [12] assumes that the volume of water associated with hydration and health in young adulthood is maintained prospectively over the life course.

“The AI for total water intake for young men and women (19 to 30 years) is 3.7 L (131 oz) and 2.7 L (95 oz)/day, respectively, which correspond to median intakes for this age group in the NHANES III survey… While it is recognized that the median intake for men and women 31 to 50 years was lower, there is no reason to assume that the level recommended for adults 19 to 30 years would be in excess. Therefore, the AI for those ages 31 to 50 years is set equal to that for younger adults…The AI for total water (drinking water, beverages, and foods) for the elderly is set based on median total water intake of young adults, rather than the older age group, in order to ensure that total water intake is not limited in the face of a potential declining ability to consume adequate amounts in response to thirst.”

[12] (pp. 145–150)

### 2.2. Alignment

In alignment with the absolute units used to quantify daily body water loss and the volume of total body water to be maintained, the NAM report expresses water intake in absolute units, ml or L/d [12] (Table 4.2, p. 80). It operationalizes water intake as total water intake (TWI), water consumed from any and/or all food and beverage sources.

“Approximately 80 percent of total water intake comes from drinking beverages and water. While consumption of beverages containing caffeine and alcohol have been shown in some studies to have diuretic effects, available information indicates that this may be transient in nature and that such beverages contribute to total water intake. While the AI is given in terms of total water, there are multiple sources of such water, including moisture from foods, beverages such as juices and milk and drinking water. While all of these can contribute to meeting the adequate intake, no one source is essential for normal physiological function and health.”

[12] (pp. 27–28)

The NAM report [12] aligns the choice of hydration biomarker with the TBW definition of hydration by selecting a biomarker that is sensitive to experimentally induced change in TBW.

“Figures 4–8 provides a compilation of 19 studies (181 subjects) where plasma osmolality was measured at several hydration levels…A strong negative relationship (*p* < 0.0001) (r = −0.76) was found between TBW changes and plasma osmolality changes… Clearly, plasma osmolality provides a good marker for dehydration status if water loss is greater than solute loss.”

[12] (p. 93)

In alignment with the assumption, noted above, that the volume of water required for hydration and health in young adulthood is maintained prospectively over the life course, the NAM report [12] treats hydrated men and women, ages 20–30 years, with no acute health condition, as the population reference groups for drawing inference from observational evidence. In alignment with the assumption that water requirements are met for each free-living individual under ad-libitum conditions of daily life, the NAM report does not control for determinants of water requirements which may vary from person to person.

### 2.3. Coordination

The NAM report [12] selects one hydration biomarker, serum osmolality, to coordinate experimental evidence about acute effects of hydration on health with observational data about the TWI of hydrated reference groups in the population. The experimental data are valid under specific controlled conditions (e.g., acute exercise induced dehydration). The observational data are generalizable to free-living conditions in the U.S.

“Tables 4–8 provides the serum osmolality for selected deciles of total water intake by gender in the Third National Health and Nutrition Examination Survey (NHANES III)… Serum osmolality concentrations were essentially identical (maximum range 3 mOsmol/kg) for the lowest (1st), middle (5th), and highest (10th) deciles within each age group. These data indicate that persons in the lowest and highest deciles of total water intake were not systematically dehydrated or hyperhydrated.”

[12] (p. 114)

“The AI for total water intake for young men and women (19 to 30 years) is 3.7 L (131 oz) and 2.7 L (95 oz)/day, respectively, which correspond to median intakes for this age group in the NHANES III survey.”

[12] (p. 145)

## 3. Potential for Alternative Methods for Gathering Evidence

Table 1 proposes alternative methods for defining, aligning, and coordinating evidence for developing water intake recommendations.

### 3.1. Alternative Definition of Hydration

While TBW deficit can imply disrupted body water distribution, as noted by the NAM report [12] (p. 99), TBW adequacy does not guarantee optimal body water distribution. Chronic health conditions, including obesity, are characterized by normal or excess TBW and an altered body water distribution, with excess extracellular relative to intracellular fluid [13]. Given that chronic disease prevention is a public health priority [11], a definition of hydration that encompasses both TBW and body water distribution may be important.

### 3.2. Alternative Assumptions

The experiments cited in the NAM report, by Adolph [14] and Newburg et al. [15], regarding ad-libitum water intake adequate to restore water lost, involve less than one week of follow-up, so do not rule out chronic TBW deficit that may take weeks to detect [16]. Working assumptions for the NAM report [12], furthermore, ignore the potential impact from known metabolic adaptations to chronic stress on long-term health [17].

### 3.3. Alternative Water Intake Measure

Beyond failing to address questions about plain water intake, specifically, the TWI measure expressed in L/d units does not account for individual differences in water requirement. The one-size-fits-all units make it difficult to tailor water intake advice to individual requirement. Clinical best practice for treating individuals for TBW deficit involves calculating the total *free water* deficit, relative to body weight in kilogram units and serum sodium, and then offering the individual *hypotonic free water* [18], i.e., plain water.

### 3.4. Alternative Biomarker of Hydration

Although continuous serum osmolality is sensitive to experimentally induced changes in TBW as well as hypertonic dehydration of cells, by itself, serum osmolality at one point in time does not provide enough information for clinical differential diagnosis and treatment planning. Clinical best practice is to evaluate both body water deficit and distribution, together, using serum sodium and urine osmolality, as biomarkers [18,19,20].

Serum sodium and osmolality have a U-shaped relationship with health risk. Serum sodium outside the normal range is associated with greater mortality [21,22,23]. To account for the U-shaped relationship, clinicians distinguish normonatremia from hypo- and hypernatremia using cutpoints such as 135 and 145 mmol/L, respectively. Rather than using serum sodium as a continuous variable for hydration assessment, as was done with serum osmolality in the NAM report [12], hydration classification should depend on categorical variables with thresholds that correspond to health risk.

### 3.5. Alternative Population Reference Groups

The population reference groups in the NAM report [12], non-acutely ill U.S. men and women, ages 20–30 years, include people who, although non-acutely ill at the time of the cross-sectional National Health and Nutrition Examination Survey (NHANES) survey, currently had or would later develop chronic health conditions. Selecting men and women who have neither acute nor chronic health conditions at ages 51–70 years removes uncertainty about chronic health condition in mid-life.

## 4. Potential for Different Evidence If Alternative Methods Are Applied

Two recent analyses of 2009–2012 NHANES data [23,24] suggest potential for alternative definitions, alignment, and coordination of evidence to overcome limitations of past methods and generate different evidence than what was gathered for the NAM report. Both analyses applied the alternative inputs described in Table 1.

### 4.1. Evidence of Need for Water

Evidence gathered using the alternative methods in Table 1 suggests that 70% or more of the non-acutely ill U.S. population, ages 19–50 years, has unmet need for water. This new evidence conflicts with evidence described in the NAM report, which suggests that essentially everyone in the non-acutely ill U.S. population adequately meets water need. According to the new data, a majority of non-acutely ill individuals in the U.S., who have serum sodium in the normal range, have urine osmolality above 500 mmol/kg [23,24]. 

Concentrated urine signals that, within the past 2–4 h, there was a need for water to offset osmotic shrinkage of cells. Concentrated urine is produced as the result of anti-diuretic hormone action, which is triggered by osmotic shrinkage of osmoreceptor cells. Although urine concentration is considered a normal homeostatic process, the need for water reflected by concentrated urine may not be benign. For adults ages 51–70 years, normal serum sodium with spot urine osmolality above 500 mmol/kg flags significantly higher risk of a current chronic health condition and death within 3 to 6 years compared to normal serum sodium with urine osmolality below 500 mmol/kg [23]. The neuroendocrine defense of body water triggered by osmotic shrinkage of osmoreceptor cells is believed to distinguish hypohydration from euhydration [25]. 

### 4.2. Evidence Regarding the Level of Water Intake Associated with Hydration and Health

Use of the alternative methods in Table 1 leads to opposite conclusions regarding how much water is associated with hydration. Citing results from NHANES III data analyses, the NAM report [12] concludes that hydration status does not vary by level of TWI for any sex or age group. Results from 2002 to 2012 NHANES data analyses, in contrast, indicate that the likelihood of meeting hydration criteria does vary significantly by level of TWI or plain water intake (PWI) for all sex and age groups [24].

Expressing water intake in ml/kg/d instead of L/d suggests different conclusions regarding how much water is associated with hydration and chronic health condition. In 2009–2012 NHANES data, for women aged 19–50 years, a greater median TWI in ml/kg is observed for women without acute or chronic health conditions than for non-acutely ill women, generally (48 mL/kg vs. 43 mL/kg) [24]. Meanwhile, TWI, expressed in absolute terms, does not appear to differ by chronic health condition. The former and latter groups have median TWI of 2.9 L/d vs. 3.0 L/d, respectively [24].

The analyses suggest that attention to chronic health condition in the selection of population reference groups may alter results. In 2009–2012 NHANES data, for men, for example, the estimated median TWI associated with meeting hydration criteria is higher by approximately 0.5 L/d, when people with chronic health conditions are included in the reference group vs. when they are excluded [24]. The median TWI for non-acutely ill men, aged 19–50 y, is 3.5 L/d, while the median TWI for non-acutely ill men in the same age group who have no chronic health conditions is 2.9 L/d [24].

The level of water intake associated with hydration and health may differ if chronic health condition and retrospective life course assumptions are applied. In 2009–2012 NHANES data, among non-acutely ill women who meet hydration criteria at ages 19–50 years, the median TWI and PWI are 43 mL/kg and 16 mL/kg, respectively [24]. The corresponding median TWI and PWI for women who are hydrated and have neither acute nor chronic health conditions at ages 51–70 years is 46 mL/kg and 23 mL/kg, respectively [24].

In contrast with the NAM report [12], which implies that PWI is not necessary to meet water requirements, the 2009–2012 NHANES data analyses [24] suggest that, for both sexes, for younger as well as middle-aged adults, hydration is associated with a median PWI over 1 L/d. In men and women, aged 19–50 years, who meet hydration criteria and do not have acute illness, the median PWI is 1.1 L/d and 1.2 L/d, respectively. For men and women at ages 51–70 years with neither acute nor chronic health conditions, the median PWI is 1.4 L/d and 1.3 L/d, respectively. The recent analysis shows that it is possible to specify water intake in terms of TWI or PWI, which means that it is theoretically possible to phrase recommendations in terms of total water from various sources OR a volume of plain drinking water [24].

## 5. Gaps in Evidence to Inform Drinking Water Recommendations for Chronic Disease Prevention

The NAM report [12] starts with experimental data, defines measures, and then commissions observational analyses to link via similar measures and units to the experimental data. Results from 2009–2012 NHANES analyses [23,24] suggest potential to work in the reverse direction, starting from the desired health outcome in nationally representative, observational data and commissioning experimental data to link with the observational data.

Nationally representative 2009–2012 NHANES data indicate that the prevalence of chronic health conditions and risk of death within 3 to 6 years is significantly lower among people who meet specified hydration criteria [23]. The data further indicate that the relative risk of meeting the same hydration criteria is significantly improved by level(s) of water intake, expressed relative to body weight, with PWI distinguished [24]. Short- and long-term experiments are lacking, however, regarding health effects of the water intake and hydration measures specified in the 2009–2012 NHANES, under controlled experimental conditions.

Beyond gaps in the literature regarding experimentally induced effects of varying levels of TWI or PWI in mL/kg and hydration (normal serum sodium with urine osmolality below 500 mmol/kg) vs. underhydration (hypernatremia and/or urine osmolality above 500 mmL/kg) on health outcomes, there are gaps in the observational and experimental literature regarding other potentially important definitions and biomarkers of hydration [e.g. 25].

## 6. Opportunity for Strategic Planning

Over the past few decades, the organic accumulation of evidence has not filled gaps in knowledge required to resolve public confusion about how much plain water is recommended for a lifetime of health in the U.S. Many data are not included in NAM reviews, such as the 2005 report [12], because they do not align or coordinate with defined standards for the review. A strategic plan is needed to guide researchers to produce evidence that will ultimately inform policy by enabling inference from the micro-level under controlled experimental conditions to the population-level under conditions of daily life. A strategic planning process offers opportunity for researchers to consider multiple possible hydration definitions, biomarkers, water intake measures, population reference groups, causal assumptions and health outcomes, before drafting a decade(s)-long plan that defines, aligns and coordinates necessary work across disciplines and contexts in a cost-efficient way.

## Figures and Tables

**Table 1:** Definition, alignment, and coordination of evidence to inform the 2005 Adequate Intake recommendation for water and possible alternative methods for future recommendations.

		Past Methods for 2005 AI [12]	Alternative Methods for Future AI
Define	Standard of evidence	RCT	RCT
	Health	Acute	Acute and Chronic
	Hydration	TBW	TBW and ECF/ICF
	Working assumptions	Chronic TBW deficit does not occur, prospective assumption that healthy in youth precedes healthy in future midlife	Chronic osmotic stress occurs and affects health, retrospective assumption that healthy in midlife implies healthy in past young adulthood
Align with definitions	Units	Absolute	Relative
	Water intake measure	TWI in L	TWI and PWI in ml/kg
	Hydration biomarker	In clinical studies, sensitive to TBW (serum osmolality)	In clinic, best practice for differential diagnosis, (serum sodium and urine osmolality)
	Reference group	Ages 20–30 years, met hydration criteria and had no acute health condition	Ages 51–70 years, met hydration criteria and had no acute or chronic health condition
	OBS data analysis	No risk factor control	Risk factor control
Coordinate	RCT data on hydration effects on health with population- representative OBS data on the level of water intake associated with hydration	Use biomarker to link	Use biomarker to link

AI: Adequate intake recommendations; RCT: Randomized clinical trial; TBW: Total body water; ECF/ICF: Extracellular: intracellular fluid ratio; TWI: Total water intake; PWI: Plain water intake; OBS: Observational
